# Selective Extraction of Nonfullerene Acceptors from Bulk-Heterojunction Layer in Organic Solar Cells for Detailed Analysis of Microstructure

**DOI:** 10.3390/ma14092107

**Published:** 2021-04-21

**Authors:** Masahiro Nakano, Akira Takahara, Kenji Genda, Md. Shahiduzzaman, Makoto Karakawa, Tetsuya Taima, Kohshin Takahashi

**Affiliations:** 1 Graduate School of Natural Science and Technology, Kanazawa University, Ishikawa 920-1192, Japan; goku0408731@gmail.com (A.T.); kenji521070@gmail.com (K.G.); karakawa@staff.kanazawa-u.ac.jp (M.K.); taima@se.kanazawa-u.ac.jp (T.T.); 2 Nanomaterials Research Institute (NanoMaRi), Kanazawa University, Ishikawa 920-1192, Japan; shahiduzzaman09@gmail.com; 3 Institute for Frontier Science Initiative (InFiniti), Kanazawa University, Ishikawa 920-1192, Japan

**Keywords:** organic solar cell, bulk-heterojunction, nonfullerene acceptor, morphology

## Abstract

Detailed analyses of the microstructures of bulk-heterojunction (BHJ) layers are important for the development of high-performance photovoltaic organic solar cells (OSCs). However, analytical methods for BHJ layer microstructures are limited because BHJ films are composed of a complex mixture of donor and acceptor materials. In our previous study on the microstructure of a BHJ film composed of donor polymers and fullerene-based acceptors, we analyzed donor polymer-only films after selectively extracting fullerene-based acceptors from the film by atomic force microscopy (AFM). Not only was AFM suitable for a clear analysis of the morphology of the donor polymers in the BHJ film, but it also allowed us to approximate the acceptor morphology by analyzing the pores in the extracted films. Herein we report a method for the selective extraction of nonfullerene acceptors (NFAs) from a BHJ layer in OSCs and provide a detailed analysis of the remaining BHJ films based upon AFM. We found that butyl glycidyl ether is an effective solvent to extract NFAs from BHJ films without damaging the donor polymer films. By using the selective extraction method, the morphologies of NFA-free BHJ films fabricated under various conditions were studied in detail. The results may be useful for the optimization of BHJ film structures composed of NFAs and donor polymers.

## 1. Introduction

Organic solar cells (OSCs) have undergone tremendous development in recent years thanks to features that have enabled them overcome the limitations found in their inorganic counterparts, the most notable of which are light weight, possible incorporation into flexible devices, and roll-to-roll processing, with the potential of low-cost mass production of power generation devices [1,2,3]. In spite of these attractive features, the practical use of OSCs remains an issue due to their low power conversion efficiency (PCE), which is a serious shortcoming in energy devices [4]. The maximum PCE in the laboratory has been steadily improving thanks to intensive investigations by many researchers [5]. Today’s OSC devices have PCEs surpassing 18% [6,7], and industrial production is expected as soon as the remaining shortcomings are overcome.

**Figure 1:**
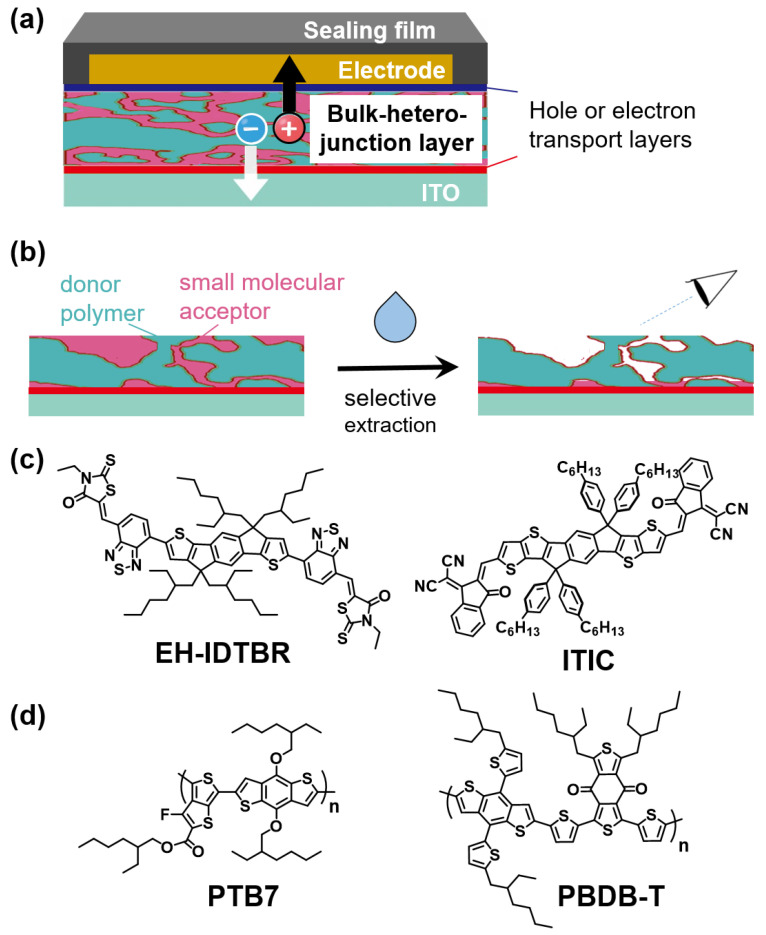
Typical structure of OPV device with BHJ layer (**a**), illustration of selective extraction method and subsequent analysis of donor/acceptor blend films (**b**), and chemical structures of NFAs (**c**) and donor polymers (**d**).

One of the important breakthroughs that has contributed to the improvement of PCEs is the utilization of a bulk-heterojunction (BHJ) structure composed of mixed donor polymers and small molecular acceptors as the active layer of OSCs, which can achieve a large donor-acceptor interface area to generate a high photocurrent (Figure 1a) [8,9,10]. The microstructure of the BHJ layer can strongly affect the photovoltaic properties. The key to improving photovoltaic performance is to develop a BHJ film with as large a donor-acceptor interface area as possible, thereby establishing pathways for electron and hole carrier transport to the upper and lower electrodes [11,12,13]. To fabricate an effective BHJ structure in films with the above features, detailed analyses of BHJ film microstructures are imperative to yield efficient OSCs with high photovoltaic performance. To this end, X-ray measurement, electron microscopy, and other special measurements are usually used. However, these analytical methods are not readily accessible, and observation of BHJ film microstructures is generally difficult. To study the BHJ layer microstructure, the selective extraction method [14,15] has been adopted, by which donor polymer-only films are obtained after the extraction of small molecular acceptors from the donor/acceptor blend films (Figure 1b). Using this method, the morphology of donor materials in BHJ films can be easily observed by atomic force microscopy (AFM). Moreover, we have approximated the morphology of acceptor materials by analyzing the pores in the extracted films [14]. We found that the selective extraction method of fullerene-based acceptors and subsequent AFM observations are effective for the detailed analysis of BHJ films and yield useful knowledge regarding BHJ films composed of fullerene-based acceptors. On the other hand, recently developed OSCs with high PCEs are composed of nonfullerene acceptors (NFAs) [16,17,18] and donor polymers with benzodithiophene units [19,20,21]. We are interested in clarifying the morphology of BHJ films in NFA-based OSCs by utilizing the selective extraction method and subsequent AFM analysis.

In this paper, we describe the AFM analysis of the microstructures of BHJ layers composed of NFAs (EH-IDTBR [22,23] and ITIC [24,25], Figure 1c) and donor polymers with a benzodithiophene unit (PTB7 [26,27] and PBDB-T [28,29], Figure 1d) after removing the NFAs by the selective extraction method. We examined utility of various organic solvents in the selective extraction method of the above materials by using donor/NFA blend films (donor/acceptor weight ratio is 1:1). Then, we studied the microstructure of the BHJ layer composed of EH-IDTBR and PTB7 by the selective extraction method and subsequent AFM analysis, which offered important knowledge in the pursuit of high photovoltaic performance.

## 2. Materials and Methods

### 2.1. Materials

We used [(5*Z*,5′*Z*)-5,5′-{[(4,4,9,9-tetra(2-ethylhexyl)-4,9-dihydro-s-indaceno[1,2-*b*:5,6-*b*’]dithiophene-2,7-diyl)bis(benzo[*c*][1,2,5]thiadiazole-7,4-diyl)]bis(methanylylidene)}bis(3-ethyl-2-thioxothiazolidin-4-one)] (EH-IDTBR) and 3,9-bis[2-methylene-{3-(1,1-dicyanomethylene)-indanone}]-5,5,11,11-tetrakis(4-hexylphenyl)-dithieno[2,3-*d*:2′,3′-*d*’]-*s*-indaceno[1,2-*b*:5,6-*b*’]dithiophene (ITIC), representative nonfullerene acceptors with good solubility, and poly[4,8-bis((2-ethylhexyl)oxy)benzo[1,2-*b*:4,5-*b*’]dithiophene-2,6-diyl-*alt*-2-((2-ethylhexyl)oxycarbonyl)-3-fluorothieno[3,4-*b*]thiophene-4,6-diyl (PTB7) and poly[(2,6-{4,8-bis[5-(2-ethylhexyl)thiophen-2-yl]benzo[1,2-*b*:4,5-*b*’]dithiophene})-*alt*-{5,5-[1´,3´-di(thiophen-2-yl)-5´,7´-bis(2-ethylhexyl)benzo[1´,2´-*c*:4´,5´-*c*´]dithiophene-4´,8´-dione]}] (PBDB-T), donor materials which are widely used for OSC devices, zinc acetylacetonate hydrate (99.995%, trace metals basis) as a precursor for the electron transporting layer film, and poly(3,4-ethylenedioxythiophene):poly(4-styrene sulfonate) (PEDOT:PSS) (Clevios P) as a material for hole transporting layer. These materials were purchased from 1-Material Inc. (Dorval, QC, Canada) and Sigma-Aldrich (St. Louis, MO, USA). The indium tin oxide (ITO) substrates (sheet resistance = 10 Ω sq) and Au (99.99%) were purchased from the Furuuchi Chemical Corporation (Tokyo, Japan).

### 2.2. Preparation of OSCs and J-V Measurements

We fabricated OSCs with an inverted structure, which can be readily fabricated and have high device durability. Indium tin oxide (ITO) electrodes on glass substrates were washed by ultrasonication in isopropanol, cleaned in boiling isopropanol, and dried by nitrogen flushing. A zinc oxide (ZnO) film was prepared by the sol-gel method reported in our previous work [30]. Zinc(II) acetylacetonate hydrate (95 mg) was dissolved in 2-methoxy ethanol (1.0 mL) containing a small amount of acetylacetone. The solution was spin-coated onto the washed ITO electrodes at 1000 rpm for 60 s. After spin coating, the precursor films were annealed for 60 min under ambient conditions. The resulting ZnO layer (ca. 30 nm) was cleaned with isopropanol. A donor/acceptor mixed chlorobenzene solution (18 mg mL^−1^) was spin-coated onto the glass/ITO/ZnO films. A PEDOT:PSS dispersion in water was spin-coated onto the active layer (1200 rpm, 60 s). Film thicknesses were ca. 100 nm for the active layer and approximately 150 nm for PEDOT:PSS. An Au back electrode of approximately 100 nm thickness was prepared by vacuum deposition at 2 × 10^−5^ Torr onto the PEDOT:PSS layer. Finally, the device covered with sealing film (Cellel, Kureha Extech) was thermo-compressed at 30 N m^−2^ for 30 s. All fabrication steps except the Au deposition step were performed in an N_2_-air mixed atmosphere to control the relative humidity to less than 30%.

The *J*-*V* characteristics of the device were measured under simulated solar illumination (AM1.5, 100 mW/cm^2^) from a solar simulator based on a 150 W Xe lamp. The light source was a SAN-EI Electric XES-40S1 solar simulator (Osaka, Japan), which was calibrated with a standard silicon photovoltaic detector. The *J*-*V* measurements were performed under ambient conditions. The active area of the device was defined by using a 1.0 cm^2^ photo mask.

### 2.3. Microstructures Sudy of BHJ Films

The sealing film, the Au electrode, and the PEDOT:PSS layer can be peeled off together from the OSC devices due to poor affinity between PEDOT:PSS and organic active materials (Supplementary Materials Figure S1). The surface of the BHJ layer was exposed by the peeling-off method. The acceptor material was extracted from the BHJ layer by slowly dropping organic solvent onto the layer and keeping the organic solvent on the layer for several minutes. Then, the organic solvent was removed by tilting the substrate (These methods were performed three times.). After careful drying in a desiccator, the resulting films were analyzed by AFM, absorption, and X-ray photoelectron spectroscopy (XPS) measurements. AFM images were measured with an SII SPI3800N AFM apparatus (Chiba, Japan). XPS analysis was performed on a Shimadzu AXIS-ULTRA DLDXPS apparatus (Kyoto, Japan). The grain and pore sizes and the number of grains/pores (pieces μm^−1^) on the extracted BHJ films were measured by using Nano Navi II software version 5.61A (SII NanoTechnology Inc., Osaka, Japan). Grain/pore measurement procedures are shown in Figure S2. Grain/pore sizes are averages of at least 50 data.

## 3. Results and Discussion

### 3.1. Selective Extraction of NFAs from Bulk-Heterojunction Films

**Figure 2:**
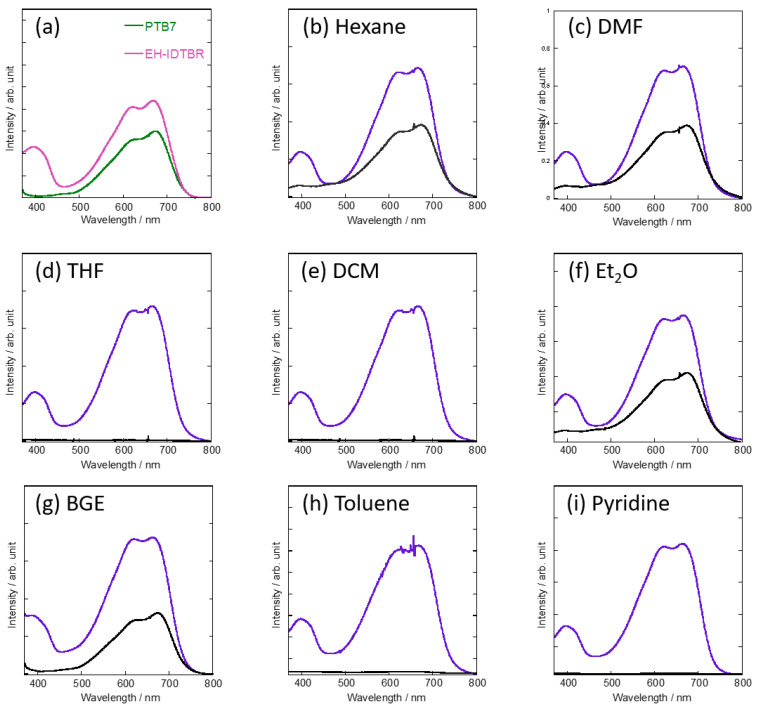
Absorption spectra of thin films of PTB7 and EH-IDTBR (**a**) and spectra obtained after extraction of EH-IDTBR from blend films (before: purple, after: black) using various organic solvents (**b**): n-hexane, (**c**): DMF, (**d**): THF, (**e**): DCM, (**f**): Et_2_O, (**g**): BGE, (**h**): toluene, (**i**): pyridine.

**Figure 3:**
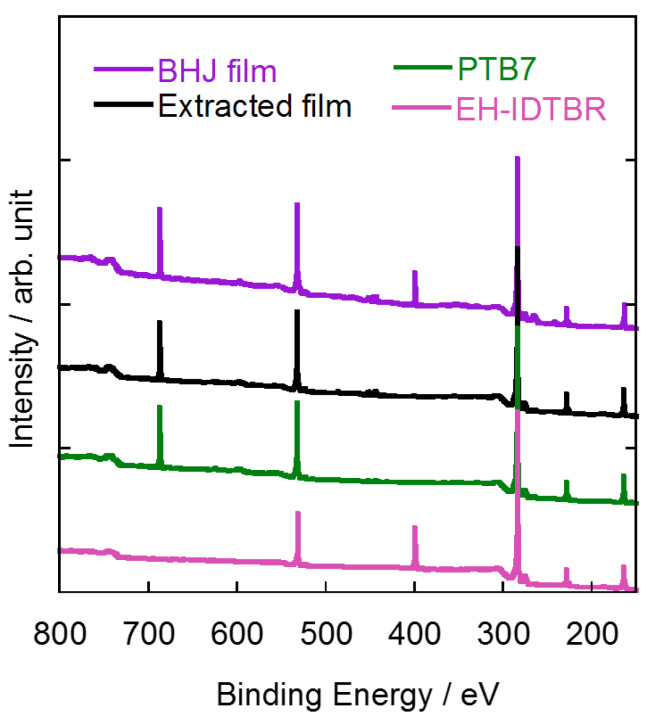
XPS spectra of BHJ films before and after extraction and of pure films of PTB7 and EH-IDTBR.

To apply the selective extraction method and the subsequent AFM observation to OSCs based on NFAs and donor polymers, we first tested various organic solvents, such as n-hexane, *N*,*N*-dimethylformamide (DMF), tetrahydrofuran (THF), dichloromethane (DCM), diethyl ether (Et_2_O), butyl glycidyl ether (BGE), toluene, and pyridine, for their ability to selectively extract EH-IDTBR from donor/acceptor blend films composed of EH-IDTBR and PTB7 ((weight of donor: D)/(total weight: D + A) = 0.50). The absorption spectra of the thin films of PTB7 and EH-IDTBR, and the blend films before and after extraction are shown in Figure 2. Thanks to high solubility of EH-IDTBR, EH-IDTBR was dissolved in all the organic solvents used in this study. However, n-hexane, DMF, and Et_2_O were not able to extract EH-IDTBR completely as suggested by the result of absorption measurements of the extracted films; the absorption spectra showed absorption at 394 nm which is the absorption of remaining EH-IDTBR (Figure 2b,c,f). On the other hand, THF, DCM, and toluene are not suitable solvents for EH-IDTBR extraction because these solvents dissolved both EH-IDTBR and PTB7 (Figure 2d,e,h). Although pyridine extracted only EH-IDTBR from the blend film, it damaged the PTB7 film and the film was peeled off from the substrate during solvent washing (Figure 2i). We found that BGE is the optimal solvent for extracting EH-IDTBR as the absorption peak at 394 nm completely disappeared after extracting EH-IDTBR (Figure 2g). The extraction of EH-IDTBR from the blend films was also confirmed by XPS measurement (Figure 3). EH-IDTBR contains nitrogen atoms, whereas PTB7 does not; thus, a thin film of EH-IDTBR showed a peak at 399.4 eV, which was assignable to 1s of nitrogen atom. The XPS spectrum of the BHJ film containing both EH-IDTBR and PTB7 also showed a peak at 399.4 eV which completely disappeared after the extraction by BGE. This suggests that EH-IDTBR was almost completely extracted from the blend films (purple and black traces in Figure 3). Moreover, using AFM, we investigated PTB7 structure in the thin films washed with BGE and compared them to the films before BGE-washing treatment. PTB7 does not dissolve in BGE, and the AFM images of PTB7 thin films before and after extraction were similar (Figure S3a,b), indicating that BGE extraction did not damage PTB7 in BHJ films. This contrasts the result of pyridine washing in which pyridine did not extract PTB7, but damaged the PTB7 film, RESULTING in a complete peeling of the film from the substrate. Moreover, PBDB-T also does not dissolve in BGE, and BGE washing did not affect the morphology of the PBDB-T thin films (Figure S3c,d), which indicated that the selective extraction of EH-IDTBR also takes place in BHJ films composed of PBDB-T. We also checked the versatility of the BGE extraction method in NFA-based BHJ films by using another representative NFA, ITIC. The absorption spectra and the XPS spectra of the blend films of ITIC and PTB7 before and after BGE washing indicated that ITIC had been extracted by BGE from the BHJ films composed of ITIC and PTB7; the absorption spectrum of the extracted film was very similar to that of the NFA-extracted film of PTB7:EH-IDTBR, and the XPS spectra showed that the peak of nitrogen atom (399.6 eV) of ITIC disappeared after BGE washing (Figure S4). Our previous study indicated that BGE is an effective solvent for extracting fullerene-based acceptors from BHJ films [14]. From these results, we can say that BGE is a useful solvent for selectively extracting not only fullerene acceptors, but also NFAs from BHJ films.

### 3.2. Microstructuer Study of BHJ Layer in OSCs Composed of NFAs

**Figure 4:**
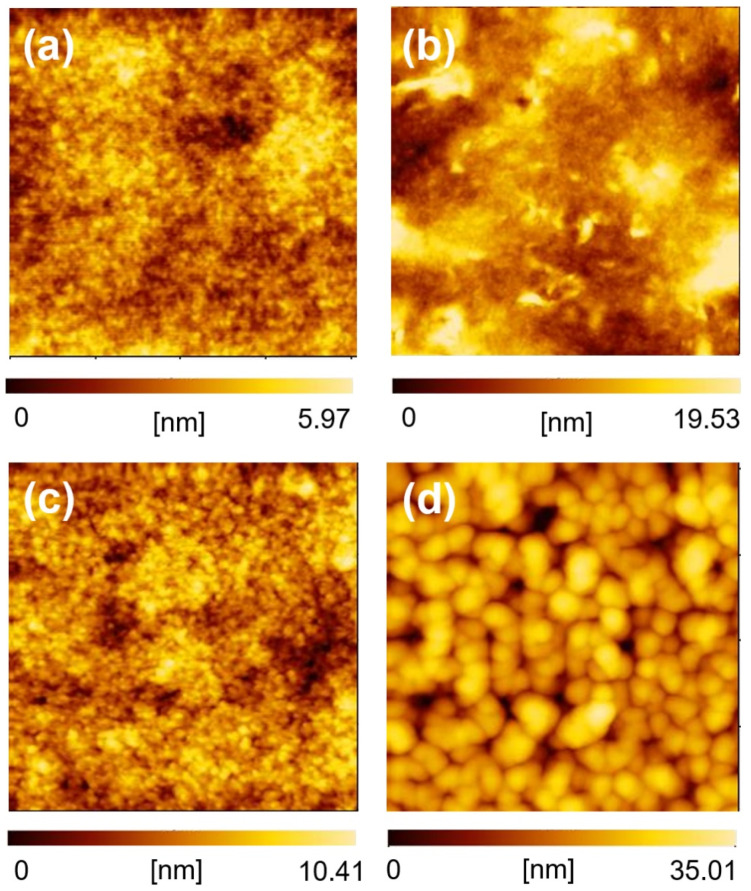
AFM images of blend films (2 μm × 2 μm) before (**a**,**b**) and after (**c**), (**d**) BGE washing. PTB7/EH-IDTBR films (**a**), (**c**) and PTB7/ITIC films (**b**,**d**).

We adopted the selective extraction method using BGE to study the microstructure of the BHJ layer in OSCs composed of NFAs (PTB7:EH-IDTBR and PTB7:ITIC, D/(D + A) = 0.50). Our experiments indicated that the selective extraction method can be applied to actual OSCs, and revealed morphological differences when different NFAs were used. Both OSCs showed similar short circuit current density (*J*_SC_) and fill factor (FF) values (Figure S5 and Table S1), as these two photovoltaic parameters are affected markedly by BHJ morphology. After the *J*-*V* measurements, the sealing film/Au/PEDOT:PSS layers were peeled off to expose the BHJ layers (PTB7:EH-IDTBR and PTB7:ITIC). Then, the acceptor materials were extracted from the BHJ layers using BGE. In contrast to the lack of distinct differences in the AFM images of the two BHJ films without BGE washing (Figure 4a,b), the AFM images of the BHJ films after BGE washing showed clear differences in the molecular aggregation of PTB7 (Figure 4c,d). PTB7 formed an amorphous-like texture in the BHJ films containing EH-IDTBR, whereas it formed clear grain-like aggregates in the BHJ films containing ITIC. The average grain size of the BHJ films extracted from PTB7:ITIC (120.1 nm) was much larger than that of the films extracted from PTB7:EH-IDTBR (67.6 nm). This implies that the phase separation nature of ITIC with PTB7 is stronger than that of EH-IDTBR with PTB7. The pore size, which is related to the segregates of the extracted acceptors, of the BGE-extracted films was also different; the pore size of the BGE-extracted PTB7:EH-IDTBR-based films was 48.1 nm and that of the BGE-extracted PTB7:ITIC-based ones was 81.6 nm. This suggests that the aggregation nature of EH-IDTBR is weaker than that of ITIC in the presence of PTB7. Moreover, the number of pores in the BGE-extracted PTB7:EH-IDTBR-based films was four times larger than that in BGE-extracted PTB7:ITIC-based films (PTB7:EH-IDTBR: 121 pieces/μm^2^ vs. PTB7/ITIC: 29 pieces/μm^2^). This indicates that the donor-acceptor interface area was larger in the PTB7:EH-IDTBR-based BHJ films than the PTB7:ITIC-based ones. Although both OSCs showed similar *J*_SC_ and FF values, their morphologies were quite different.

### 3.3. Morphological Study of BHJ Films in Optimization of OSC Fabrication Conditions

**Figure 5:**
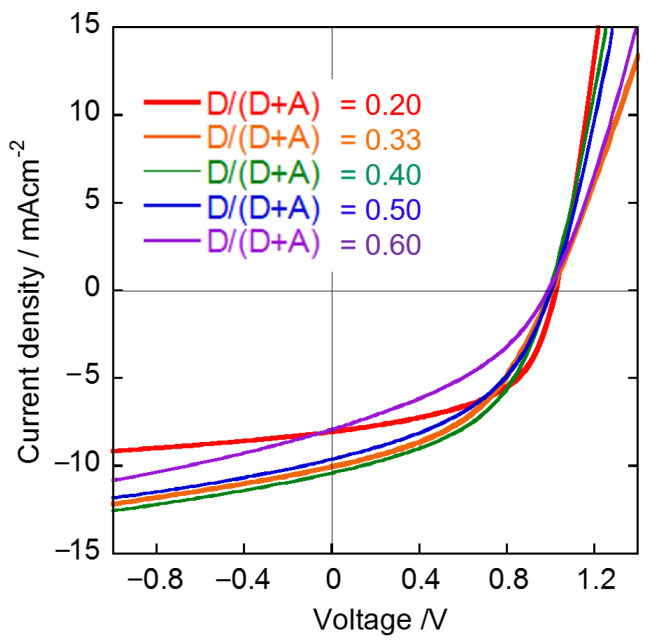
*J*-*V* characteristics of OSC devices composed of PTB7:EH-IDTBR.

**Figure 6:**
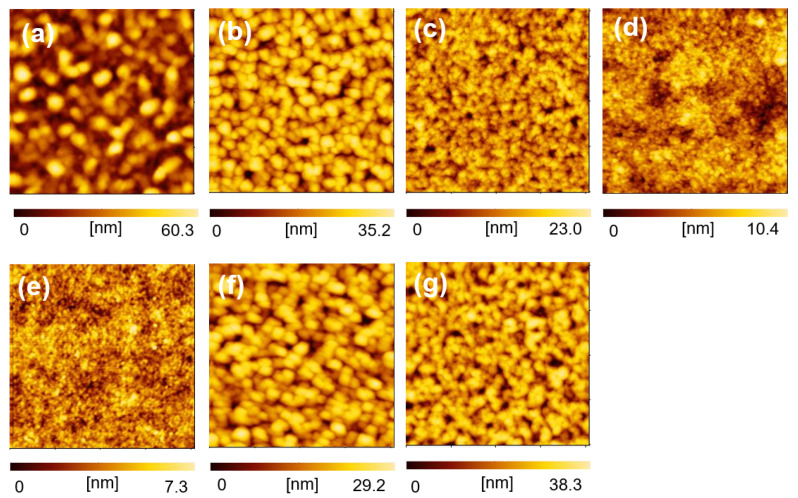
AFM images of BHJ films composed of PTB7:EH-IDTBR after BGE washing (2 μm × 2 μm). D/(D + A) ratios of PTB7:EH-IDTBR are as follows: (**a**) 0.20, (**b**) 0.33, (**c**) 0.40, (**d**) 0.50, (**e**) 0.60, (**f**) 0.33 with 1 vol% nitrobenzene, and (**g**) 0.33 with 2.5 vol% nitrobenzene.

**Figure 7:**
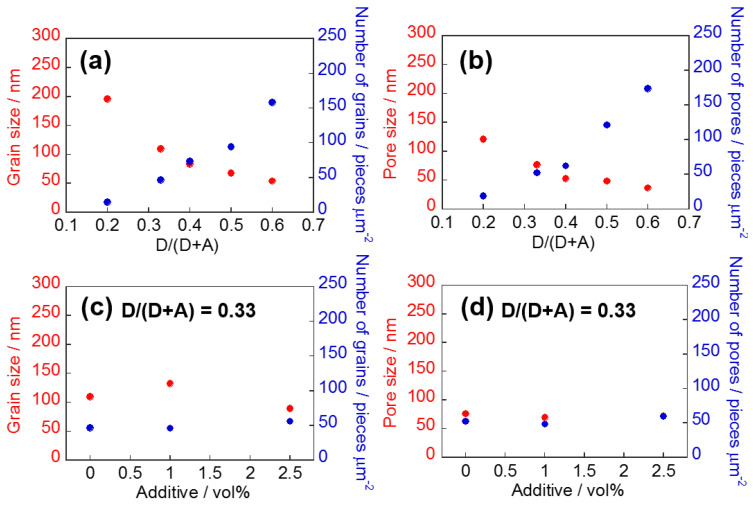
Dependence on D/(D + A) ratio (D: donor, A: acceptor) of grain/pore size (averaged values) and number of grains/pores (pieces μm^−2^) (**a**,**b**). Dependence on additive amount of grain/pore size and number of grains/pores (**c**,**d**).

In OSC studies, optimization of photovoltaic properties is generally conducted by applying various techniques for morphology control of the BHJ layer. We focused on the morphological study of BHJ films composed of NFAs by using the selective extraction method and AFM analysis, because knowledge of morphological changes during device optimization could be useful for device physics. One of the general methods for the optimization of OSCs is the optimization of donor/acceptor weight (D/(D + A)) ratio in a mixed solution for BHJ film fabrication, which can affect the morphology of the BHJ films and the resulting photovoltaic properties. We studied the morphological changes and photovoltaic properties of the BHJ films using the selective extraction method and AFM analysis when D/(D + A) ratio was changed. OSC devices composed of PTB7:EH-IDTBR with different D/(D + A) ratios (0.20, 0.33, 0.40, 0.50, and 0.60) were fabricated and their photovoltaic properties were measured. Then, the morphologies of the BHJ films were observed after exposure of the BHJ films with BGE washing. As shown in Figure 5 and Table 1, OSCs with excess amounts of donor or acceptor materials (D/(D + A) = 0.20 and 0.60) have low *J*_SC_ and PCE values (PCE: <4.0%), and OSCs with D/(D + A) ratios of 0.33 and 0.40 have good photovoltaic properties (PCE: 4.6–4.7%). On the other hand, the morphology of the remaining PTB7 films after BGE washing is dependent on the D/(D + A) ratio (Figure 6a–e). The analyzed result of the grain and pore sizes and their numbers are shown in Figure 7. The fact that the grain sizes are larger than the pore sizes implies that PTB7 has a stronger aggregation nature than EH-IDTBR. As the D/(D + A) ratio of PTB7 increased, the grain and pore sizes decreased. This implies that PTB7 and EH-IDTBR tend to aggregate under an environment where there are many EH-IDTBRs, whereas PTB7 and EH-IDTBR mix well under an environment where there are many PTB7s.

We were interested in PTB7:EH-IDTBR-based OSCs with D/(D + A) ratios of 0.20 and 0.60 because these devices showed similar *J*_SC_ and different FF values (*J*_SC_: 7.75 and 7.93 mA cm^−2^, FF: 0.46 and 0.37). As both parameters were decreased by the “poor” morphology of the BHJ films, detailed analysis and comparison of these cases would offer important knowledge. The grain/pore sizes of the remaining BHJ films with the D/(D + A) weight ratio of 0.60 (BHJ^0.60^) are smaller and the numbers of grains/pores are larger than those of the remaining BHJ films with the D/(D + A) ratio of 0.20 (BHJ^0.20^). For BHJ^0.60^-based OSCs, the carrier transport path would be limited and the dissociation of hole/electron carriers would occur frequently in the BHJ layers due to the large number of grains/pores in BHJ^0.60^. This can make the resulting FF poor [31]. For BHJ^0.20^-based OSCs, the photocurrent was much lower than the ideal case due to the large grains/pore sizes of donor-acceptor materials. This could originate in the larger grain/pore radius than the exciton diffusion length of NFAs (for EH-IDTBR: 10–20 nm, for ITIC: 20–30 nm) [32] and donor polymers [33]. However, this did not affect FF. The results suggest that we may be able to predict the mechanism underlying the poor photovoltaic properties by using the selective extraction method and AFM analysis in detail.

Considering the photovoltaic properties, the suitable D/(D + A) ratio of the OSCs for photocurrent generation is 0.33 to 0.40 (PCE: 4.6–4.7%), at which the photovoltaic parameters are similar and the *J*_SC_ and FF values are slightly different. Such differences in photovoltaic performance are caused by subtle differences in the morphology of the BHJ films. From the photovoltaic properties, we predict that the suitable grain size is 83–109 nm and the suitable pore size is 52–76 nm to obtain high PCE values. Although these grain/pore radii are larger than the diffusion length, the suitable grain/pore size should be determined by a balance of carrier transport path and donor-acceptor interface area. Another typical technique for controlling BHJ morphology is the use of additives in active material solutions for BHJ film fabrication. Using the additives, we can obtain different morphological changes when the D/(D + A) ratio is optimized. We used nitrobenzene to further optimize of BHJ^0.33^ morphologies and found that PCE was improved (5.2%) when 1 vol% of nitrobenzene was used. AFM analysis after the selective extraction method revealed that the addition of nitrobenzene did not change pore size and number markedly (Figure 7d). On the contrary, grain size was changed; the addition of 1 vol% of nitrobenzene increased grain size (109 nm to 132 nm), whereas the addition of 2.5 vol% of nitrobenzene decreased grain size (89 nm). This indicates that nitrobenzene affects the aggregation of PTB7 without changing the aggregation of EH-IDTBR, and the 132 nm grain size of PTB7 is suitable to achieve high photovoltaic performance PTB7:EH-IDTBR-based OSCs. These findings for the BHJ films of PTB7 and EH-IDTBR would contribute to the optimization of OSCs based on those materials.

## 4. Conclusions

In this paper, we reported the application of the selective extraction method to OSCs based on NFA and a state-of-the-art donor polymer with benzodithiophene units. We found that BGE is an effective solvent for the selective extraction of BHJ films composed of EH-IDTBR, ITIC, PTB7, and PBDB-T, as revealed by absorption and XPS measurements. After the selective-extraction method, the detailed morphology of the BHJ films was clarified by AFM measurement. Although morphological changes in BHJ films composed of similar materials (PTB7-Th and EH-IDTBR) under various conditions were studied [34], we were able to examine the morphology in detail without using special measuring equipment. The results indicate that the selective extraction method and subsequent AFM analysis can be used for device analysis and optimization of NFA-based OSCs to overcome the low PCEs of OSCs. Further investigation and development of nonfullerene OSCs using the selective extraction method are underway in our laboratory.

## Figures and Tables

**Table 1:** Summarized photovoltaic performances.

D/(D + A) ^a^	Additive/Vol% ^b^	*J*_SC_/mA cm^−2^	*V*_oc_/V	FF	PCE/%
0.20		7.75 ± 0.23	0.99 ± 0.02	0.46 ± 0.02	3.46 ± 0.26
0.33		10.17 ± 0.17	0.99 ± 0.01	0.48 ± 0.02	4.67 ± 0.29
0.40		10.33 ± 0.21	0.99 ± 0.01	0.46 ± 0.01	4.57 ± 0.29
0.50		9.37 ± 0.25	1.00 ± 0.01	0.43 ± 0.01	4.01 ± 0.22
0.60		7.93 ± 0.19	0.94 ± 0.01	0.37 ± 0.00	2.71 ± 0.04
0.33	1	10.35 ± 0.20	0.99 ± 0.01	0.51 ± 0.02	5.20 ± 0.29
0.33	2.5	9.78 ± 0.10	0.99 ± 0.01	0.45 ± 0.01	4.31 ± 0.08

^a^ D: weight of donor material, A: weight of acceptor material; ^b^ additive: nitrobenzene.

## Data Availability

The data presented in this study are available on request from the corresponding author. The data are not publicly available due to the huge amount of data.

## Supplementary Materials

The following are available online at https://www.mdpi.com/article/10.3390/ma14092107/s1, Figure S1: Peeling-off of Sealing film/Au/PEDOT:PSS from the OSC devices, Figure S2: Outline for estimating grain/pore size from AFM images of an acceptor-extracted blend film, Figure S3: AFM images (2 μm × 2 μm) of thin films of PTB7 before (a) and after (b) BGE washing and of thin films of PBDB-T before (c) and after (d) BGE washing, Figure S4: Absorption spectra of blend films of ITIC and PTB7 before and after extraction with BGE (a), and XPS spectra of ITIC film and blend film extracted with BGE (b), Figure S5: *J*-*V* characteristics of PTB7:EH-IDTBR-based OSC and the PTB7:ITIC-based OSC (D/(D + A) = 0.50), Figure S6: EQE spectra of PTB7:EH-IDTBR-based OSCs (D/(D + A) = 0.50), Figure S7: Changes of PCE of the OSC device based on PTB7:EH-IDTBR (D/(D + A) = 0.50) under continuous photo-irradiation (AM1.5G, 100 mWcm−2, ambient conditions, 120 min), Table S1: Summarized photovoltaic properties of OSCs based on PTB7:EH-IDTBR and PTB7:ITIC (D/(D + A) = 0.50).
