# Supplementary material for: Selective Extraction of Nonfullerene Acceptors from Bulk-Heterojunction Layer in Organic Solar Cells for Detailed Analysis of Microstructure
Source: Materials (Basel). 2021 Apr 21;14(9):2107. doi: 10.3390/ma14092107 (PMC8122272; doi:10.3390/ma14092107)
Supplement: Supplementary file 1 [file materials-14-02107-s001.zip › materials-1181774-supplementary.pdf]

Supplementary Materials

Selective Extraction of Nonfullerene Acceptors from Bulk-Heterojunction Layer in Organic Solar Cells for Detailed Analysis of Microstructure

S1. Peeling-off of Sealing Film/Au/PEDOT:PSS from the OSC Devices

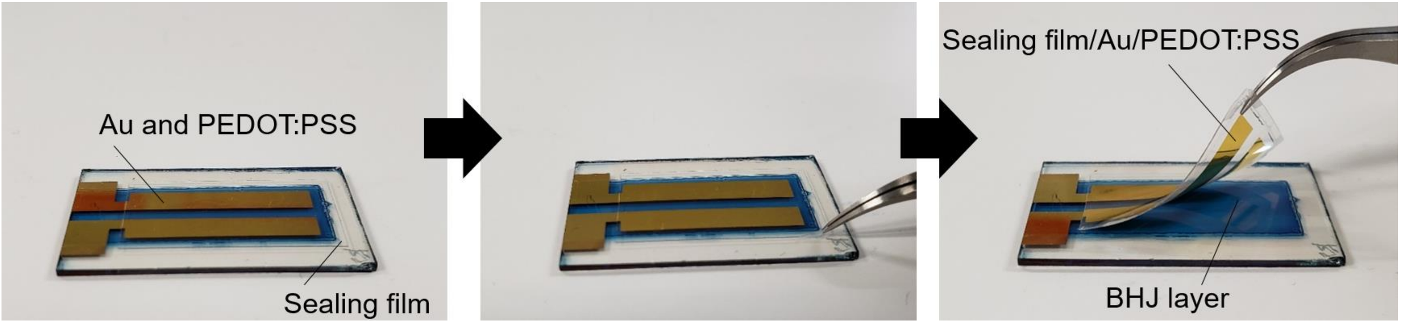

Figure S1. Peeling-off of Sealing film/Au/PEDOT:PSS from the OSC devices.

S2. Outline for Estimating Grain/Pore Size from AFM Images

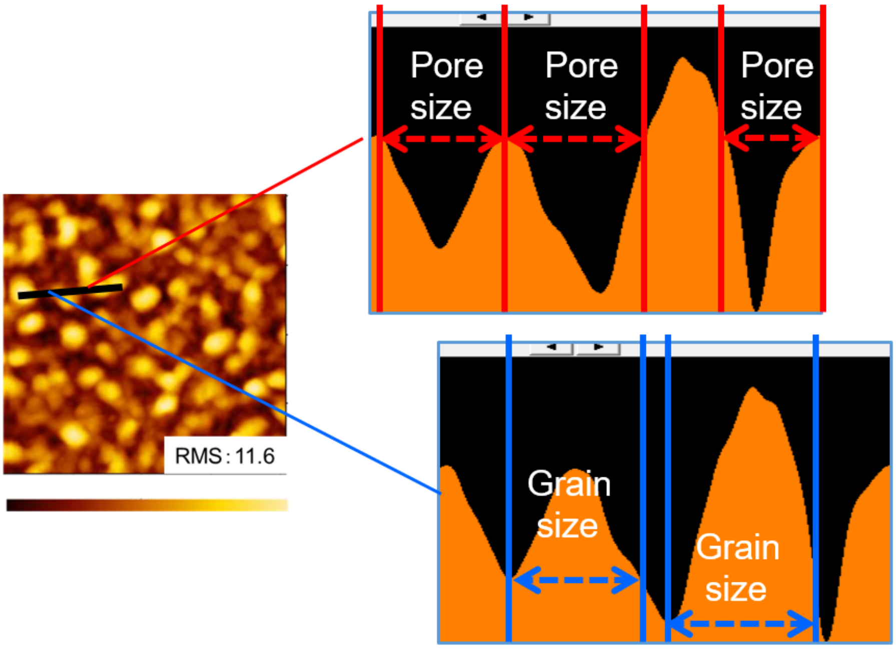

Figure S2. Outline for estimating grain/pore size from AFM images of an acceptor-extracted blend film.

S3. AFM Images of Thin Films of PTB7 and PBDB-T before and after BGE Washing

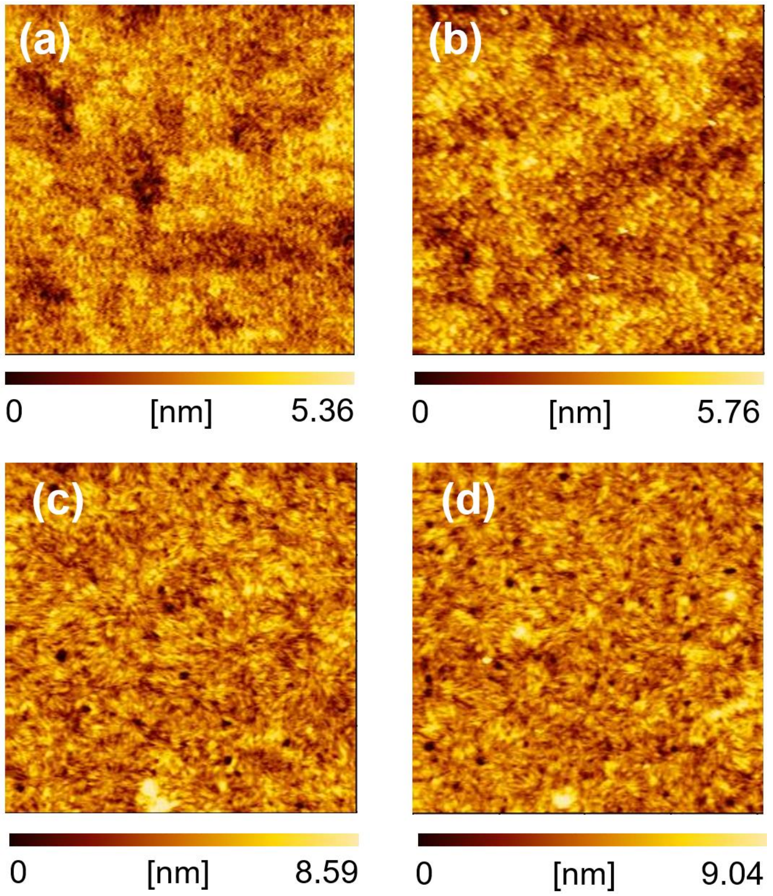

Figure S3. AFM images (2 µm × 2 µm) of thin films of PTB7 before (**a**) and after (**b**) BGE washing and of thin films of PBDB-T before (**c**) and after (**d**) BGE washing.

S4. Absorption and XPS Spectra of Blend Films of ITIC and PTB7 before and after Extraction

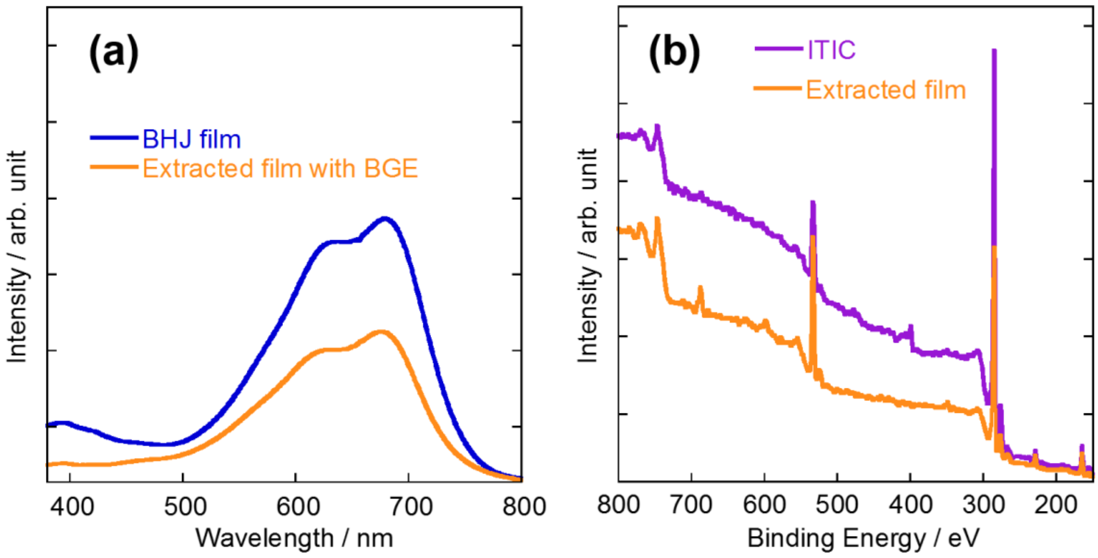

Figure S4. Absorption spectra of blend films of ITIC and PTB7 before and after extraction with BGE (**a**), and XPS spectra of ITIC film and blend film extracted with BGE (**b**).

S5. Photovoltaic Properties of OSCs Based on PTB7:EH-IDTBR and PTB7:ITIC

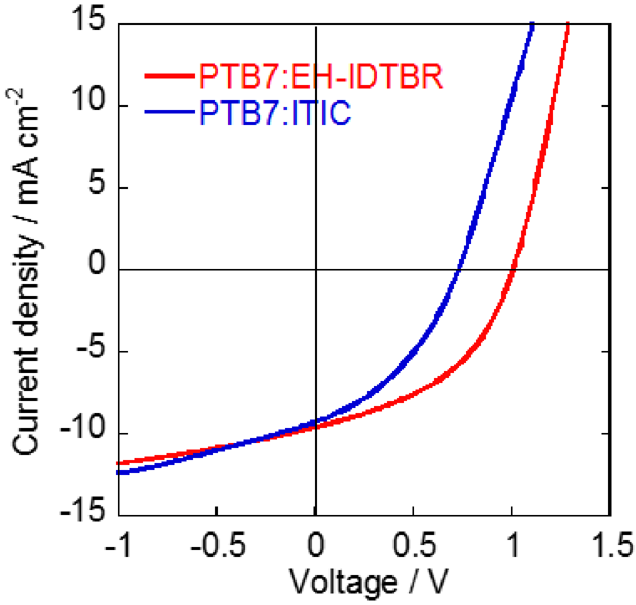

Figure S5. *J-V* characteristics of PTB7:EH-IDTBR-based OSC and the PTB7:ITIC-based OSC (D/(D + A) = 0.50).

Table S1. Summarized photovoltaic properties of OSCs based on PTB7:EH-IDTBR and PTB7:ITIC (D/(D + A) = 0.50).

acceptor	J_{SC} / mA cm^{-2}	V_{OC} / V	FF	PCE / %
EH-IDTBR	9.37(±0.25)	1.00(±0.01)	0.43(±0.01)	4.00(±0.20)
ITIC	9.13(±0.32)	0.74(±0.01)	0.40(±0.01)	2.64(±0.05)

S6. EQE Spectra and Stability Testing of PTB7:EH-IDTBR-Based OSCs

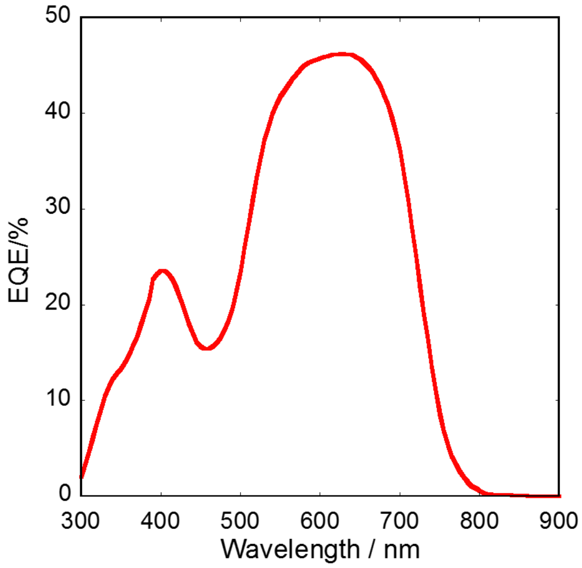

Figure S6. EQE spectra of PTB7:EH-IDTBR-based OSCs (D/(D + A) = 0.50).

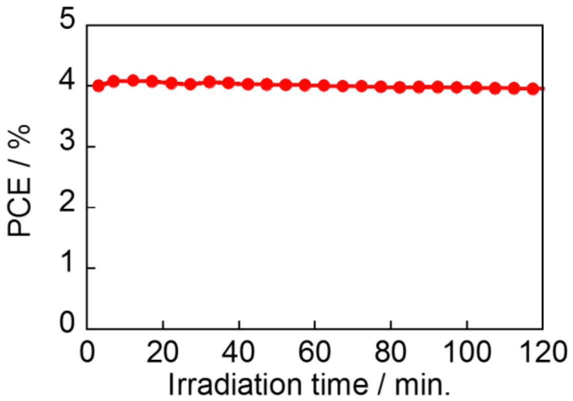

Figure S7. Changes of PCE of the OSC device based on PTB7:EH-IDTBR (D/(D + A) = 0.50) under continuous photo-irradiation (AM1.5G, 100 mWcm^{-2}, ambient conditions, 120 min).
